# Arsenic Exposure and the Western Diet: A Recipe for Metabolic Disorders?

**DOI:** 10.1289/ehp.124-A39

**Published:** 2016-02-01

**Authors:** Julia R. Barrett

**Affiliations:** Julia R. Barrett, MS, ELS, a Madison, WI–based science writer and editor, is a member of the National Association of Science Writers and the Board of Editors in the Life Sciences.

Chronic arsenic exposure is common in many areas worldwide owing to naturally occurring contamination of well water.^1^ Arsenic has been shown to contribute to various cancers, skin lesions, and cardiovascular disease.^1^ Epidemiological studies on arsenic and metabolic outcomes such as nonalcoholic fatty liver disease, obesity, and diabetes have yielded mixed results, however,^2^^,^^3^^,^^4^^,^^5^ although regional variations in factors such as diet could explain the discrepancies. A new mouse study in this issue of *EHP* suggests that prenatal and early-life exposures to low-level arsenic, combined with a Western-style diet, may induce developmental changes that heighten the risk of future metabolic disorders and nonalcoholic fatty liver disease.^6^

A small body of evidence indicates that prenatal and early-life arsenic exposures can influence the development of later disease.^1^^,^^7^^,^^8^ For instance, studies of Chilean adults who were exposed to high levels of arsenic prenatally and as very young children had unusually high death rates from lung disease and heart attack.^8^ “This really gave the evidence that developmental [arsenic] exposure, even just *in utero* or fetal exposure alone, could lead to consequences in adulthood,” says Todd Camenisch, an associate professor of pharmacology and toxicology at the University of Arizona, who coauthored the current study.

**Figure:**
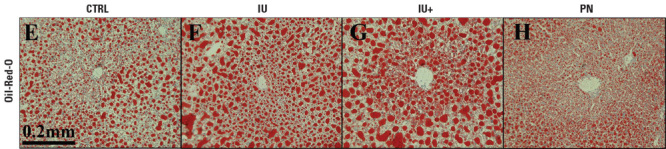
Staining with a dye called Oil Red O shows increased lipid accumulation in the livers of mice exposed prenatally to arsenic (IU and IU+), compared with unexposed mice (CTRL) and those exposed only postnatally (PN). In humans, increased lipid accumulation is a risk factor for cardiometabolic disease. Ditzel et al. (2016)^6^

In previous research Camenisch and colleagues studied metabolic disease risk in mice exposed prenatally to low-level arsenic.^9^ In those experiments, offspring of female mice that drank water containing 100 ppb sodium arsenite during pregnancy developed nonalcoholic fatty liver disease and other signs of heightened risk for metabolic syndrome, a constellation of symptoms associated with diabetes and cardiovascular disease in humans.

The current study built on that work by testing how diet might affect metabolic disease risk in animals exposed to low levels of arsenic. To start, one group of pregnant mice was given water containing 100 ppb sodium arsenite. Half their offspring discontinued arsenic exposure at birth (the *in utero*, or IU, treatment group), while the other half continued exposure (the IU+ treatment group). A second group of pregnant mice received untreated water; half their offspring drank water containing sodium arsenite after weaning (the postnatal, or PN, treatment group), while the other half served as the untreated control group.

All offspring were weaned to a high-fat, high-sugar Western-style diet and weighed weekly. Lipid and glucose metabolism blood tests were conducted at weaning (3 weeks of age), at 5 weeks, and at 9 weeks. At 13 weeks the livers of all offspring were examined for histologic changes, gene expression, lipid content, and enzymatic activity. Male offspring had significantly higher weight gain, so the researchers focused on them for the current analysis.

The results showed that IU and IU+ exposures exacerbated nonalcoholic fatty liver disease in the mice. Disruptions were seen in energy metabolism, specifically impaired glucose control, insulin resistance, increased obesity, and increased blood levels of triglycerides. Effects were not as severe in the IU group as for the IU+ mice, but they were still more prominent than in the PN and control groups.^6^ “Overall, we were sort of surprised that we had such disruptions,” says Camenisch.

Studies such as this that are designed to identify critical windows of exposure (e.g., prenatally versus postnatally) can help inform effective policies to prevent exposures; dose is another important consideration. Gavin Arteel, a professor of pharmacology and toxicology at the University of Louisville, says one particular strength of this study was the focus on arsenic concentrations that are relevant to human exposure, although including animals on a low-fat diet for comparison might have brought differences into even sharper focus. Arteel, who was not involved in the study, says, “It’s certainly a proof of concept, though, and that’s another strength.”
